# Distinct Mutation Signatures in Peripheral Blood Mitochondrial DNA from Liquid Biopsy Reveal Insights into Pancreatic Cancer

**DOI:** 10.3390/cells15060527

**Published:** 2026-03-16

**Authors:** Hannah Randeu, Abel Bronkhorst, Angela Oberhofer, Karolina Worf, Carsten Uhlig, Eleni Polatoglou, Zsuzsanna Mayer, Klara Dorman, Danmei Zhang, Stefan Boeck, Volker Heinemann, Michael Haas, Stefan Holdenrieder

**Affiliations:** 1 Department of Internal Medicine III and Comprehensive Cancer Center, Klinikum Grosshadern, Ludwig-Maximilians-University Munich, 81377 Munich, Germany; hannah.randeu@gmail.com (H.R.); klara.dorman@med.uni-muenchen.de (K.D.); danmei.zhang@med.uni-muenchen.de (D.Z.); stefan.boeck@med.uni-muenchen.de (S.B.); volker.heinemann@med.uni-muenchen.de (V.H.); 2 German Cancer Consortium (DKTK), Partner Site Munich, 80336 Munich, Germany; 3 Munich Biomarker Research Center, Institute of Laboratory Medicine, German Heart Center, Technical University Munich, 80636 Munich, Germany; abel.bronkhorst29@gmail.com (A.B.); a.oberhofer@tum.de (A.O.); karolina.worf@tum.de (K.W.); c.uhlig@tum.de (C.U.); polatoglou@dhm.mhn.de (E.P.); mayerz@dhm.mhn.de (Z.M.); 4 Center for the Evaluation of Biomarkers, CEBIO GmbH, 81679 Munich, Germany; 5 Department of Hematology and Oncology, München Klinik Neuperlach, 81737 Munich, Germany

**Keywords:** pancreatic cancer, liquid biopsy, mtDNA, personalized medicine, NGS

## Abstract

**Highlights:**

**What are the main findings?**

- Whole-blood mtDNA profiling revealed pancreatic cancer-associated differences in mutation allele frequency (AF), regional distribution, and heteroplasmy, despite similar overall mtDNA mutational burden between patients and healthy controls.
- Increased variability in mtDNA copy number and specific mtDNA features (high-AF variants, unique SNV patterns) were associated with differences in overall survival, possibly reflecting systemic mitochondrial stress rather than tumor-intrinsic mutations alone.

**What are the implications of the main findings?**

- Blood-derived mtDNA captures a systemic mitochondrial signal shaped by tumor presence, immune responses, and oxidative stress, providing complementary biological insight beyond tissue-based analyses.
- Integrated mtDNA features may support the development of minimally invasive models for risk assessment, patient stratification, and disease monitoring in pancreatic cancer, even in the absence of a single definitive biomarker.

**Abstract:**

Pancreatic cancer (PC) is a highly aggressive malignancy characterized by limited opportunities for early diagnosis and poor clinical outcomes, underscoring the need for minimally invasive biomarkers to improve detection and patient stratification. Given emerging evidence that mitochondrial DNA (mtDNA) alterations reflect cancer-related biological processes, this study investigated whether blood-derived mtDNA profiles could provide clinically relevant information in PC. In this exploratory study, whole-blood mtDNA from 33 PC patients and 10 healthy individuals were analyzed using next-generation sequencing to assess single-nucleotide variants (SNVs), allele frequencies, and mtDNA copy number. A total of 252 unique mtDNA SNVs were identified, including variants exclusive to PC patients, variants unique to controls, and variants shared between groups. While the overall SNV burden did not differ significantly between groups, PC patients showed distinct mutation distributions and allele frequency patterns, with cancer-exclusive variants occurring predominantly at low allele frequencies. Mutation hotspots were observed in the ND5, COI, and D-loop regions, implicating genes involved in oxidative phosphorylation and mtDNA maintenance. Although mtDNA copy number did not differ significantly between groups, greater variability was observed among PC patients and was associated with differences in survival outcomes. Overall, these findings indicate that blood-based mtDNA profiling captures biologically relevant variation associated with PC and supports further development of integrated mtDNA-based approaches for improved risk assessment and patient stratification.

## 1. Introduction

Pancreatic cancer (PC) is a highly aggressive malignancy and the third leading cause of cancer-related deaths in the United States [1]. Its incidence is rising rapidly [2,3,4,5], and is projected to become the second leading cause of cancer mortality worldwide by 2030 [6,7,8]. Despite advances in cancer biology, PC remains difficult to diagnose and treat. Over 50% of patients are diagnosed at an advanced stage, with poor prognosis and a 5-year survival rate that remains dismally low [9,10]. Incidence and mortality rates are nearly identical, reflecting disease aggressiveness and limited therapies, and although surgical resection is potentially curative, it is feasible only in early-stage disease, underscoring the need for early detection [2]. However, no effective screening methods exist for PC [11], and the widely used diagnostic marker CA 19-9 lacks sensitivity in early stages and can be elevated in non-cancerous conditions [12]. The urgent need for novel biomarkers to facilitate earlier detection and guide treatment strategies is clear.

Mitochondrial DNA (mtDNA) has emerged as a promising cancer biomarker, with aberrations linked to oncogenesis, disease progression, multiple cancer types, and increased metastatic potential [13,14,15,16,17,18,19,20]. Characterizing mtDNA mutations in PC may inform novel diagnostic and therapeutic strategies, as mitochondria regulate apoptosis, immunity, metabolism, and ROS homeostasis, and their dysfunction is a hallmark of many diseases [21,22,23,24]. Unlike nuclear DNA, mtDNA is more prone to mutations due to the absence of protective histones, introns, and robust repair mechanisms [25]. Consequently, mtDNA mutations occur more frequently and can impair oxidative phosphorylation in cancer cells [21,26]. Tumor-associated metabolic alterations suggest that pathogenic mutations, heteroplasmy, and changes in mtDNA copy number may serve as potential cancer biomarkers [27,28,29,30]. Mitochondria play a central role in the tumor microenvironment, which is shaped by immune infiltration, hypoxia, and metabolic reprogramming [31,32]. Because the tumor microenvironment can suppress immune responses and promote progression [33,34], mtDNA alterations in whole blood, largely derived from immune cells, may reflect tumor aggressiveness and treatment resistance and could represent potential therapeutic targets [35]. mtDNA is maternally inherited and typically homoplasmic, but mixed mutant and wild-type variants, termed heteroplasmy, are common even in healthy individuals and vary across tissues [36,37,38]. While heteroplasmic mutations are often asymptomatic, functional impairment can occur once a critical threshold is exceeded or when mutations become homoplasmic [39]. Given the high copy number of mtDNA per cell, its analysis may offer increased sensitivity, particularly in liquid biopsy approaches [40].

Liquid biopsy is an emerging clinical tool that enables minimally invasive analysis of tumor DNA from blood, supporting real-time and longitudinal monitoring of PC and other malignancies. In this context, mtDNA analysis via liquid biopsy is a promising avenue for investigating mitochondrial dysfunction in PC, with potential to improve diagnostic accuracy and provide novel therapeutic insights. Next-generation sequencing (NGS) facilitates comprehensive mitochondrial genome profiling, including detection of single nucleotide variants (SNVs), heteroplasmy assessment, and mtDNA copy-number quantification in a cost-effective and scalable manner [41,42,43,44].

Most prior studies have examined mtDNA from tumor tissue or plasma to capture tumor-intrinsic alterations or cell-free DNA released during cell death, whereas whole-blood mtDNA primarily reflects mitochondrial genomes from circulating blood and immune cells exposed to systemic inflammation, oxidative and metabolic stress, and cancer therapy, potentially providing a more stable measure of chronic systemic mitochondrial remodeling. Whether whole-blood mtDNA captures cancer-associated systemic or immune processes remains unclear. In this study, we analyzed whole-blood mtDNA from healthy individuals and patients with PC, assessing SNVs, base substitution patterns, regional distribution across the mitochondrial genome, heteroplasmy levels, and mtDNA copy number, and exploring associations with clinical characteristics and outcomes, including overall survival (OS). By characterizing qualitative features of whole-blood mtDNA variation, we aimed to determine whether it conveys biologically and clinically relevant information beyond tumor-derived mutations and may support earlier detection, biomarker development, patient stratification, and improved monitoring within personalized medicine approaches.

## 2. Materials and Methods

### 2.1. The Subjects and Ethical Considerations

Whole-blood samples were obtained from 10 healthy individuals with no history of PC or other malignancies and from 33 PC patients. Collection of samples from the healthy cohort was approved by the ethics committee of Ludwig-Maximilians-Universität (LMU) München (Project No. 21-0707) as part of a previously approved study [28]. The PC cohort included 8 samples from the RASH study (n = 150) and 10 samples from the ACCEPT study (n = 119), both recruiting patients with metastatic PC [45,46], as well as 17 samples from the Alpaca study. All participants provided informed consent for the use of their blood samples. Alpaca patients were treated at LMU and enrolled in the project “The Informative Patient” (project number 284-10; ethics approval obtained).

### 2.2. Sample Collection and mtDNA Extraction

A total of 8.5 mL blood was collected by venipuncture in PAXgene™ Blood DNA tubes (PreAnalytiX GmbH, Hombrechtikon, Switzerland) and stored at −80 °C. MtDNA was isolated and quantified as previously described [28].

### 2.3. Library Preparation and Sequencing

MtDNA samples were prepared for sequencing using the QIAseq Targeted DNA Panel, Human Mitochondria Panel Library Preparation Kit (Qiagen, Hilden, Germany) according to the manufacturer’s protocol. Briefly, DNA was fragmented, end-repaired, A-tailed, and ligated with adapters containing combinatorial dual indices for multiplexing. Libraries then underwent target enrichment and PCR amplification to selectively enrich mtDNA fragments. Library quantity and quality were assessed using the Qubit dsDNA HS Assay, QIAseq Library Quant Assay Kit (Qiagen), and the Bioanalyzer High Sensitivity DNA Kit (Agilent Technologies, Santa Clara, CA, USA). Sequencing was performed on the NextSeq 2000 platform (Illumina, San Diego, CA, USA) following the manufacturer’s instructions, with libraries converted to molarity and diluted appropriately prior to loading.

### 2.4. Data Analysis

Raw sequencing reads underwent quality control using FastQC [47] v0.11.9, fastp [48] v0.23.2, and MultiQC [49] v1.15.dev0 to assess sequencing quality and detect potential issues. Adapter trimming (adapter sequence: CAAAACGCAATACTGTACATT), quality filtering, and removal of sequencing artifacts were performed with fastp using standard settings, including paired-end adapter detection (--detect_adapter_for_pe) and front-end trimming for read2 (-F25). High-quality reads were aligned to the mitochondrial genome of the human reference GRCh38, including HLA sequences, using Burrows–Wheeler Aligner (BWA) [50] v0.7.17-r1188 with default parameters. SAM files were converted to BAM, name-sorted, mate-fixed, coordinate-sorted, indexed, and summarized using Samtools [51] v1.13; duplicate reads were removed using samtools markdup -r. Per-base coverage across the mitochondrial genome was calculated using samtools depth -a, including positions with zero coverage, and all samples showed consistently high and uniform coverage without systematic dropout. Variant calling and heteroplasmy estimation were performed using the MitoHPC pipeline [52] (version 20230427) with its standard workflow (Mutect2 caller, two-iteration consensus approach, and heteroplasmy thresholds of 3%, 5%, and 10%). A minimum read-depth filter of DP ≥ 100 was applied, and variants flagged by the predefined MitoHPC filtering rule (HP_FRULE), including strand-bias or homopolymer-associated artifacts, were excluded. All preprocessing, alignment, and variant-calling steps were performed using default or recommended settings and applied uniformly across all samples without study-specific parameter optimization. *Estimated mitochondrial copy number* was calculated in R Statistical Software [53] v4.4.0 using the *average coverage* derived from Samtools coverage files, the size of the *human mitochondrial genome* in GRCh38 (16,569 bp), and an *assumed copy number* constant (n = 100), as follows:

$$estimated\;copy\;number=\frac{average\;coverage\;*\;mitochondrial\;genome\;size}{assumed\;copy\;number}$$

The constant value of 100 was introduced as a fixed computational scaling factor to facilitate comparability across samples and does not represent a biological assumption of 100 mtDNA copies per cell. Rather, this approach converts sequencing depth into relative mtDNA abundance under uniform analytical conditions. Accordingly, the resulting values reflect comparative differences between samples and should not be interpreted as absolute mtDNA copies per cell or total cellular mtDNA content.

### 2.5. Statistical Analysis and Limitations

Statistical analyses and data visualization were performed using GraphPad Prism version 9 (GraphPad Software, San Diego, CA, USA). Data were presented using box plots, violin plots, pie charts, bar graphs, and scatter plots, as appropriate. Box plots display mean values with standard deviation unless otherwise specified, while violin plots illustrate the distribution and density of individual data points. Pie charts summarize proportional distributions of categorical variables, bar graphs depict counts or normalized frequencies, and scatter plots were used to evaluate linear associations between variables.

This study was designed as an exploratory, hypothesis-generating analysis including 33 patients and 10 controls; no a priori power calculation was conducted. Post hoc estimates indicate that, for two-group comparisons (two-sided α = 0.05, 80% power), the study is primarily powered to detect large, standardized effects (minimum detectable Cohen’s d ≈ 1.0). Therefore, non-significant findings do not exclude moderate or small differences, including in case–control (n = 33 vs. 10) and within-patient subgroup analyses (n = 15 vs. 18). Survival analyses are additionally constrained by small subgroup sizes and event numbers; detectable hazard ratios are approximately 2.7–3.2 under near-complete follow-up and would be higher with censoring. Accordingly, survival-related findings should be considered hypothesis-generating and warrant validation in larger, independent cohorts. Additional methodological considerations are discussed in Supplementary File S4.

## 3. Results and Discussion

### 3.1. Analysis of mtDNA SNVs Between Cancer Patients and Healthy Individuals

**Figure 1:**
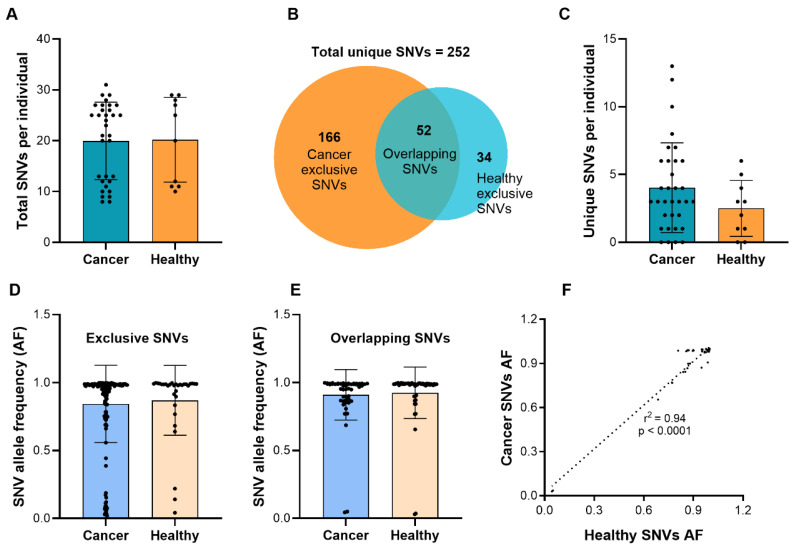
Distribution of mtDNA SNVs across PC patients (n = 33) and healthy controls (n = 10). (**A**) Comparison of the total number of SNVs per individual between cancer and healthy groups, showing no significant difference. (**B**) Venn diagram illustrating the overlap of unique SNVs between cancer and healthy groups. A total of 252 unique SNVs were identified, with 166 being exclusive to cancer, 34 exclusive to healthy individuals, and 52 shared between both groups. (**C**) Number of unique SNVs per individual for cancer and healthy groups, showing a higher number of unique SNVs in PC patients compared to healthy individuals. (**D**) AF of exclusive SNVs for cancer and healthy individuals, showing a similar distribution across both groups. (**E**) AF of overlapping SNVs between cancer and healthy individuals, again showing similar AFs. (**F**) Strong correlation (r^2^ = 0.94, *p* < 0.0001) between the AFs of overlapping SNVs in cancer and healthy samples, suggesting a consistent SNV pattern across conditions. Given n = 33 vs. n = 10, between-group comparisons are powered primarily for large effects; moderate or small differences may not reach statistical significance and cannot be excluded. No statistically significant differences (ns) are present in these comparisons.

We first compared the total number of mtDNA SNVs per individual between cancer patients and healthy controls and observed no significant difference (Figure 1A), indicating a comparable overall mtDNA mutational burden in whole blood. Thus, total SNV count alone did not distinguish cancer from non-cancer states, and subsequent analyses focused on qualitative features of mtDNA variation. Because mtDNA was isolated from whole blood rather than tumor tissue or plasma, the detected variants predominantly reflect blood cell-derived mtDNA; the contribution of tumor-derived mtDNA cannot be determined. Accordingly, the observed alterations represent systemic mtDNA variation associated with cancer rather than a tumor-intrinsic mitochondrial mutational landscape. In this context, “cancer-exclusive” SNVs refer to variants detected only in PC patients and absent in healthy controls, without implying causality or functional relevance.

**Figure 2:**
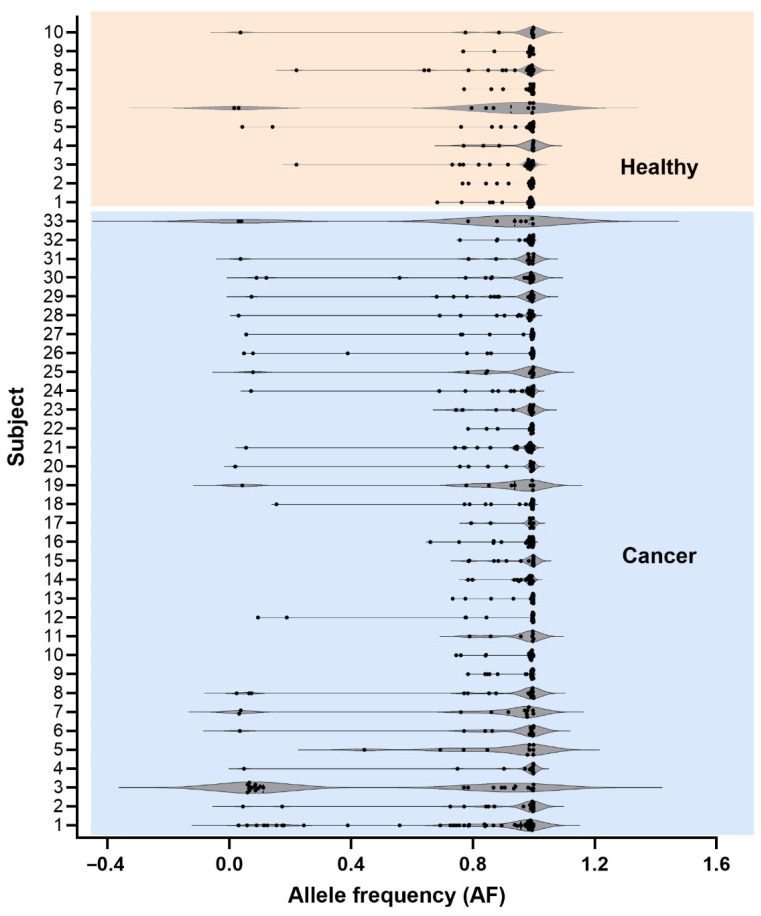
Distribution of AFs of mtDNA SNVs in healthy (n = 10) and cancer subjects (n = 33). Each horizontal line corresponds to an individual subject, with the top section (highlighted in orange) representing healthy individuals and the bottom section (highlighted in blue) representing PC patients. Black dots indicate the AFs of individual mtDNA SNVs for each subject, while the surrounding violin plots illustrate the distribution, spread, and density of these frequencies. The data reveal notable differences in the AF distributions between healthy and cancer subjects, suggesting potential associations between mtDNA variation and disease status.

Across the cohort, 252 unique SNVs were identified: 166 exclusively in PC patients, 34 exclusively in healthy individuals, and 52 shared between groups (Figure 1B). PC patients harbored more unique SNVs per individual on average (Figure 1C), largely driven by a subset with elevated variant counts. Healthy individuals showed fewer exclusive variants, consistent with reports of relatively stable mtDNA mutation patterns under physiological conditions [54]. To distinguish categorical from global patterns, AF dynamics were examined from complementary perspectives. Figure 1D,E present AF distributions stratified by variant class, specifically cancer-exclusive and overlapping SNVs. Although overall AF distributions are broadly comparable between groups, cancer-exclusive SNVs exhibit lower average AFs than overlapping variants (Figure 1D), indicating enrichment at lower heteroplasmy levels. Overlapping SNVs show strongly correlated AFs between PC patients and controls (r^2^ = 0.94, *p* < 0.0001; Figure 1F), demonstrating consistent frequency patterns across groups. Figure 2, in contrast, evaluates AF distributions across all detected SNVs at the individual level, independent of variant classification. This analysis reveals substantial inter-individual variability in both groups.

### 3.2. Base Substitution Distribution of mtDNA Mutations in Cancer and Healthy Tissues

**Figure 3:**
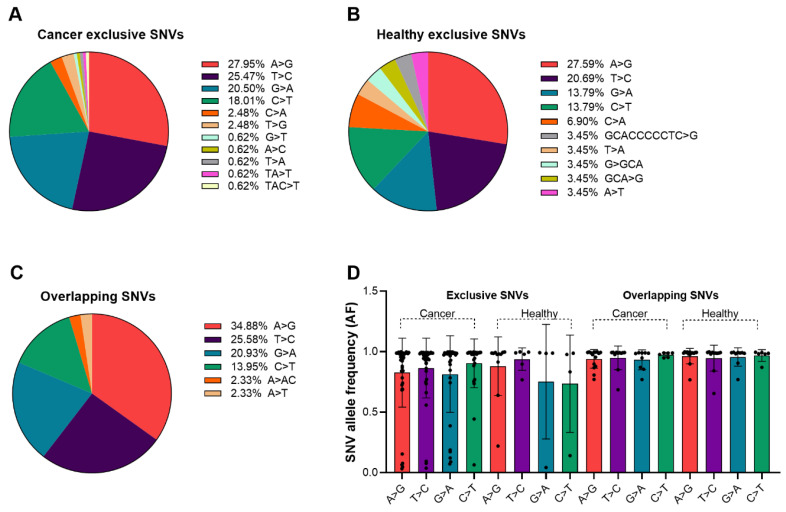
Base substitution distribution of mtDNA mutations. (**A**) Pie chart depicting the distribution of base substitutions among cancer-exclusive mtDNA SNVs (n = 166). The most frequent base changes observed were A>G (27.95%) and T>C (25.47%), with smaller contributions from other mutation types. (**B**) Pie chart showing the base substitution distribution of healthy-exclusive mtDNA SNVs (n = 34). (**C**) Pie chart illustrating the base substitution distribution of overlapping mtDNA SNVs (n = 52), shared between both cancer and healthy individuals. (**D**) Bar graph comparing the AFs of specific base substitutions across exclusive SNVs in cancer and healthy individuals, as well as overlapping SNVs shared between both groups. No statistically significant differences were observed in AF between different base substitution types. Error bars indicate standard deviation.

We analyzed base substitution patterns of mtDNA SNVs within the previously defined variant categories (Figure 3A–C). In cancer-exclusive SNVs, defined as variants detected only in PC patients, A>G (27.95%) and T>C (25.47%) were most frequent. Healthy-exclusive SNVs showed a similar predominance of A>G (27.59%) and T>C (20.69%), with a broader distribution of less common substitutions (Figure 3B). Overlapping SNVs exhibited comparable patterns, with A>G (34.88%) and T>C (25.58%) predominating (Figure 3C). Overall, transition mutations, particularly A>G and T>C, dominated across all categories, consistent with established mitochondrial and nuclear DNA mutation spectra [55,56]. G>A transitions accounted for 20% of substitutions in PC patients compared to 14% in healthy individuals, whereas other substitution types, including C>T transitions and G>C or T>G transversions, occurred at lower frequencies in both groups. The relative enrichment of G>A substitutions in PC patients may reflect cancer-associated biological processes, including mitochondrial alterations in activated immune cells, as previously described [32,33,34,57], although further investigation is required. Substitution patterns commonly linked to exogenous carcinogens were not prominent, consistent with reported mitochondrial genomic features such as high copy number and quality-control mechanisms that influence mutation spectra [58,59,60]. While Figure 2 presents global AF dynamics, Figure 3D evaluates AF distributions both across all SNVs and stratified by substitution category. AF distributions within defined base substitution classes showed comparable heteroplasmy ranges across groups, indicating no evident enrichment of substitution types among low-frequency SNVs in this dataset.

### 3.3. Region Wise Distribution and Landscape of mtDNA SNVs

**Figure 4:**
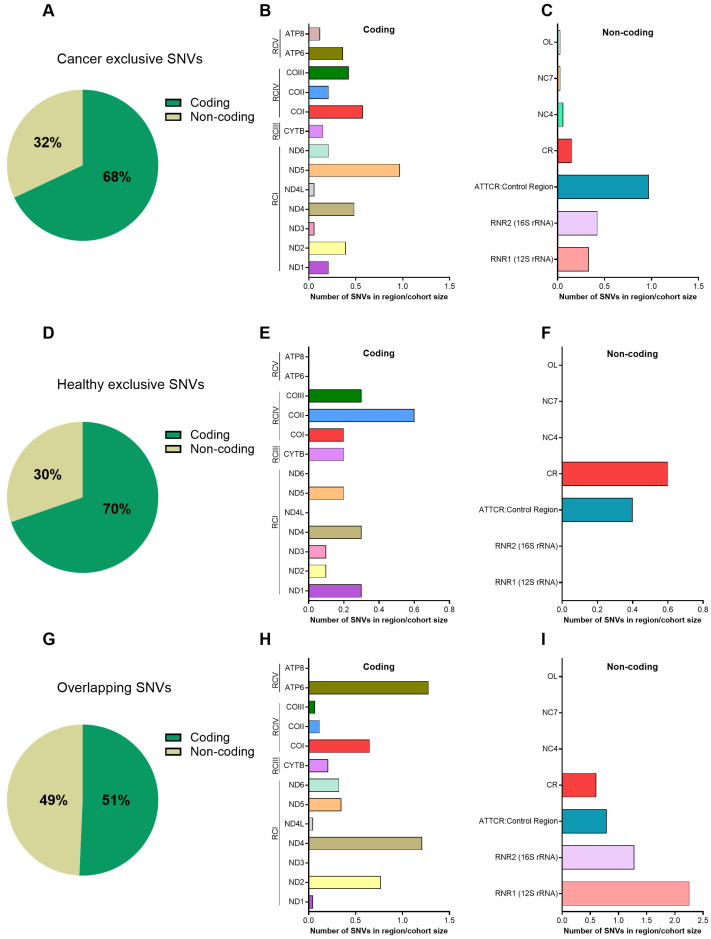
Distribution of mtDNA SNV frequencies across coding and non-coding regions. SNVs are categorized as cancer-exclusive (detected only in PC patients, n = 33), healthy-exclusive (detected only in healthy controls, n = 10), or overlapping (detected in both cohorts, n = 43). (**A**–**C**) Cancer-exclusive SNVs, (**D**–**F**) healthy-exclusive SNVs, and (**G**–**I**) overlapping SNVs. (**A**,**D**,**G**) Pie charts showing the proportion of coding versus non-coding SNVs. (**B**,**E**,**H**) Bar charts showing coding-region SNVs. (**C**,**F**,**I**) Bar charts showing non-coding-region SNVs. The *y*-axis represents mtDNA regions, and the *x*-axis represents the total number of SNVs normalized by cohort size (i.e., total SNVs divided by 33, 10, or 43, respectively).

We first examined the regional distribution of mtDNA SNVs to determine whether specific genomic compartments were enriched (all data in Supplementary File S1). Cancer-exclusive (found only in PC patients) and healthy-exclusive SNVs were predominantly located in coding regions, with approximately 70% mapping to coding and 30% to non-coding regions (Figure 4A,D). In contrast, overlapping SNVs were more evenly distributed between coding and non-coding regions (approximately 50% each; Figure 4G). At the gene level, cancer-exclusive SNVs were enriched in ND5 and COI (Figure 4B), encoding subunits of respiratory chain Complex I and IV, respectively, and showed a lower mutational burden in healthy controls (Figure 4E). ND5 displayed the most pronounced increase in PC patients. Previous studies have linked ND5 mutations to mitochondrial dysfunction, increased ROS production, genomic instability, and activation of oncogenic pathways such as EGFR signaling [59,61,62,63,64,65,66]. Non-coding mutations were also increased in PC samples, particularly in the control region containing the D-loop (Figure 4C vs. Figure 4F), a known regulatory hotspot for mtDNA replication and transcription that is frequently altered in multiple cancers [67]. Its increased mutational burden in PC is consistent with reports associating D-loop alterations with mitochondrial dysfunction and aging [60,68,69]. Overlapping SNVs were enriched in ATP6, ND4, ND2, and the ribosomal RNA genes RNR1 and RNR2 (Figure 4G–I), distinct from regions enriched for exclusive variants. This distribution was largely driven by common mtDNA polymorphisms (Table 1), including chrM:1438 A>G and chrM:8860 A>G, which occurred at high frequency in both groups and are consistent with population-level variation.

**Figure 5:**
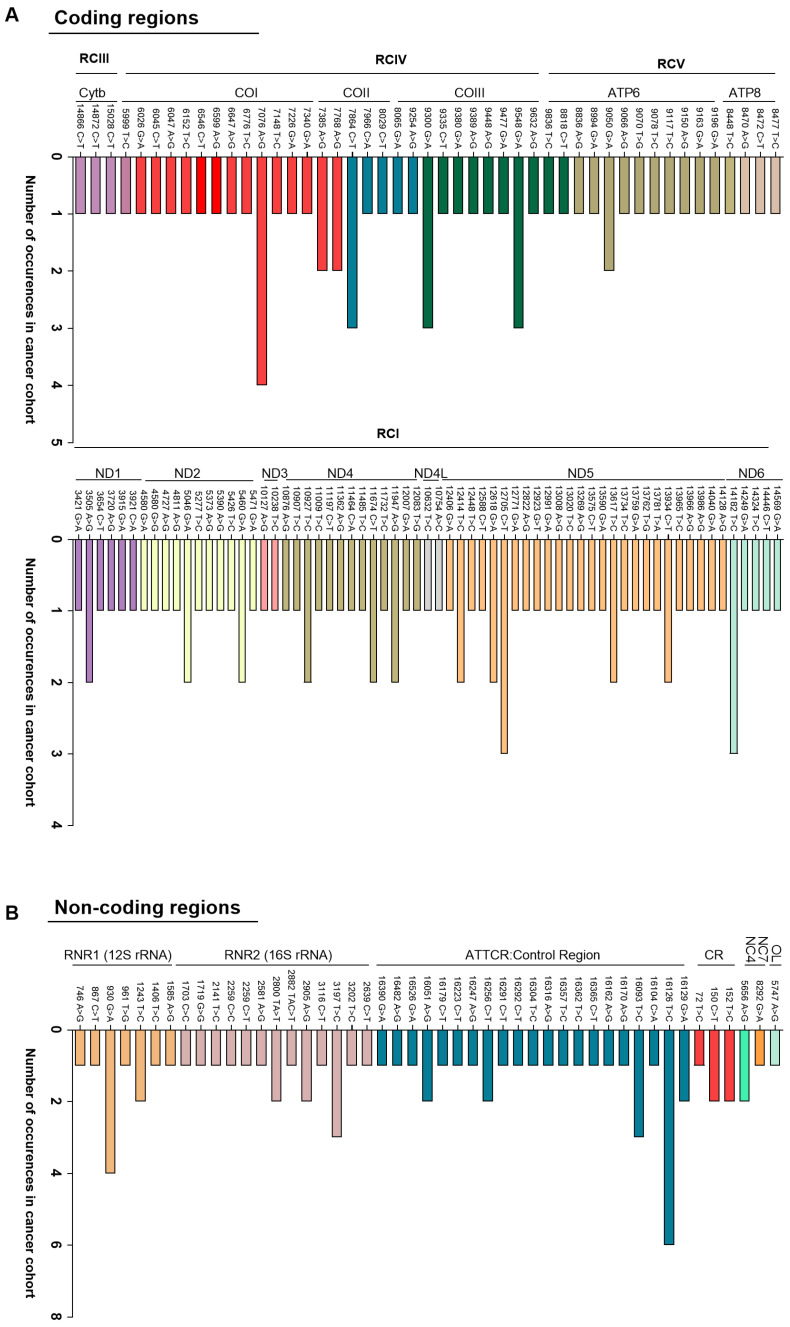
Landscape of mtDNA SNVs found exclusively in PC patients (n = 33). (**A**) Coding regions: Distribution of mtDNA SNVs across mitochondrial coding regions, grouped by mitochondrial genes. (**B**) Non-coding regions: Distribution of cancer-exclusive mtDNA SNVs identified in non-coding regions. The *x*-axis represents the frequency of each SNV across the cancer cohort. Each bar corresponds to a specific SNV, with bar height indicating the number of patients in whom the SNV was observed, and distinct colors representing different mitochondrial regions or genes.

We next examined the specific SNVs within these regions to map the mutational landscape and identify recurrent or potentially novel variants (Figure 5), all SNVs summarized in Supplementary File S2. Coding-region variants frequently affected COI, COII, ATP6, and ND1–ND6. Recurrent mutations included 7076 A>G and 7884 T>C in COI and multiple variants in ND5 observed in up to four patients. These genes encode key components of the oxidative phosphorylation pathway and have previously been implicated in tumor biology [15,20,26,70]. Cross-referencing with MITOMAP and published datasets identified 57 variants previously reported, including 19 described in PC cell lines. Overall, 95% of SNVs were exclusive to our PC cohort. Among coding-region mutations unique to PC patients, recurrent variants included 7076 A>G (COI); 9548 G>A and 9300 G>A (COIII); 7864 C>T (COII); 14182 T>C (ND6); and 12705 C>T (ND5). Of these, only 12705 C>T in ND5 has previously been associated with cancer, including gastric, hepatocellular, renal cell, and prostate malignancies [15,70,71,72]. In non-coding regions, 16126 T>C in the D-loop was exclusive to PC patients and detected in five of six cases at near-homoplasmic levels (AF > 0.99). Although not previously reported in PC, this variant has been described in glioblastoma and breast cancer [73,74].

### 3.4. Correlations Between Mutational Signatures and Clinical Parameters

All available clinical characteristics of the PC patients, together with a complete overview of all recorded variables, are provided in Supplementary File S3. Associations between mtDNA-derived variables and clinical characteristics were assessed using linear correlation analyses, subgroup stratification, and targeted group comparisons. Clinical parameters included age, sex, EGFR therapy status, and OS. Relative mtDNA copy number and SNV-related features were examined in relation to these variables to explore potential associations between mitochondrial genomic characteristics and clinical outcome. Important prognostic variables, including disease stage, treatment line, tumor burden, performance status, comorbidities, risk factors (e.g., smoking status and obesity), and the timing of blood sampling relative to therapy, were not consistently available. Their absence limited adjustment for potential confounding and precluded multivariable modelling. Consequently, associations between mtDNA features and clinical outcomes may partly reflect underlying clinical heterogeneity. All clinical correlations should therefore be considered exploratory and hypothesis-generating. In light of these limitations and to avoid overinterpretation, potential clinical associations are discussed in detail in Supplementary File S4, with supporting figures in Supplementary File S5, whereas only a condensed summary is presented here in the main text.

Mean plasma mtDNA copy number did not differ significantly between PC patients and healthy controls. However, variability was greater among patients, indicating substantial interindividual heterogeneity (Supplementary File S4, Figure S1A). No linear correlation was observed between mtDNA copy number and SNV AF. Nevertheless, patients with higher mtDNA copy number showed a greater incidence of high-AF SNVs and shorter OS than those with lower mtDNA copy number (Supplementary File S4, Figure S1C,D). No significant differences were observed with respect to age, total SNV count, unique SNV count, or cancer-specific SNV (those only found in the cancer cohort) count. Because mtDNA copy number was estimated from sequencing coverage without normalization to nuclear DNA, these findings should be regarded as exploratory and interpreted with caution.

Total mtDNA SNV count was not significantly associated with the major clinical variables examined. However, it correlated with the number of unique SNVs, cancer-specific SNVs, and most strongly with variants also detected in healthy controls (Supplementary File S5, Figure S4A–C). Patients with more unique SNVs tended to have longer OS and more high-AF SNVs, although the association with survival was sensitive to exclusion of a single outlier and should therefore be interpreted cautiously (Supplementary File S4, Figure S2A–C). Stratification by AF showed that the high-AF patient group had a greater proportion of cancer-specific SNVs, whereas mtDNA copy number did not differ significantly between AF groups (Supplementary File S4, Figure S2D–E). Stratification according to cancer-specific SNV abundance did not reveal significant clinical differences, although unique and cancer-specific SNVs showed partial overlap within the cohort (Supplementary File S5, Figure S4D,E).

Age was not significantly associated with any of the clinical parameters analyzed. Male patients exhibited significantly higher heteroplasmy than female patients (Supplementary File S5, Figure S4H, whereas no other significant sex-related differences were observed.

No consistent linear relationship was identified between OS and the clinical variables assessed. However, stratified analyses suggested that higher mtDNA copy number was associated with shorter OS, whereas a greater abundance of unique SNVs tended to be associated with longer OS (Supplementary File S4, Figures S1D and S2B). Patients with shorter OS also tended to be older, although this difference did not reach statistical significance (Supplementary File S4, Figure S3A,B). Given the exploratory design, small subgroup sizes, and absence of multivariable modelling, these findings require validation in larger cohorts.

## 4. Summary and Conclusions

This study characterizes mtDNA variation in whole blood from patients with PC and healthy controls. Because mtDNA was isolated from whole blood rather than tumor tissue or plasma, detected SNVs primarily reflect mtDNA from circulating blood cells and differ from tumor-intrinsic or cell-free mtDNA profiles reported previously. Accordingly, these alterations represent peripheral blood mtDNA variation and cannot be directly attributed to tumor-specific mitochondrial mutations. Across samples, both previously reported and numerous unreported or rarely described mtDNA SNVs were detected. Although total mtDNA SNV counts did not differ between groups, PC was associated with distinct mutational patterns, AF distributions, and region-specific enrichment. These findings indicate that qualitative mtDNA mutation features, rather than total mutational burden, provide more informative biological and clinical insights in this dataset.

Shared mtDNA SNVs between PC patients and controls showed highly concordant AFs, indicating that much mtDNA variation reflects common polymorphisms or background variation. In contrast, several SNVs found only in the cancer cohort were low-AF variants, suggesting less clonally expanded events in circulating blood cells. Their biological origin cannot be determined and may reflect tumor-derived DNA, systemic cancer-associated processes, treatment effects, or inflammation. Independent validation, ideally including matched tumor and plasma samples, will be required to confirm disease associations and clarify clinical relevance. Mutational signature analysis showed a transition-dominated mtDNA spectrum in both groups, with relative enrichment of G>A transitions in PC patients. Although compatible with oxidative damage, this pattern is not specific to a defined mechanism. Region-based analyses showed clustering of SNVs found only in the cancer cohort in ND5, COI, and the D-loop, whereas overlapping SNVs were enriched in ATP6, ND2, ND4, and ribosomal RNA genes, largely reflecting known polymorphisms. SNVs found only in the cancer cohort and healthy-exclusive SNVs were mainly located in coding regions (≈70%), with the remainder in non-coding regions, whereas overlapping SNVs were distributed more evenly between coding and non-coding regions (≈50% each). The identification of near-homoplasmic D-loop variants such as 16126 T>C, together with previously unreported mtDNA mutations, expands the descriptive catalog of blood-derived mtDNA variation observed in this cohort. Given the exploratory design and limited sample size, larger studies are needed to determine whether mtDNA variant diversity or AF patterns relate to clinical outcomes in PC.

Clinically, mean mtDNA copy number did not differ significantly between groups, although variability was greater among PC patients. Higher relative mtDNA copy number was associated with enrichment of high-AF variants and shorter OS in exploratory subgroup analyses, indicating statistical associations within this dataset rather than mechanistic links to disease aggressiveness or mitochondrial stress. Conversely, patients with a broader spectrum of unique mtDNA SNVs tended to have longer OS, whereas cancer-specific SNV burden alone was not predictive of outcome. Male patients exhibited higher heteroplasmy levels than female patients without differences in OS, indicating sex-related variation in mtDNA features without demonstrated prognostic impact.

Overall, whole-blood mtDNA profiling identified reproducible differences in mitochondrial genomic variation between PC patients and healthy individuals in this cohort, with qualitative mtDNA features rather than total mutation burden distinguishing groups in this dataset. However, given the exploratory design, modest cohort size, absence of matched tumor samples, relative mtDNA quantification, and subgroup analyses sensitive to outlier exclusion, these findings remain descriptive and hypothesis-generating. Larger, well-annotated cohorts with longitudinal sampling and matched tumor and nuclear DNA normalization will be required to determine biological origin, validate clinical associations, and assess potential clinical utility.

## Figures and Tables

**Table 1:** Common SNVs found in both PC patients and healthy controls.

Region	Mutation	Cancer Frequency of Occurrence	Healthy Frequency of Occurrence
12S	chrM:1438 A>G	33	10
12S	chrM:750 A>G	31	10
16S	chrM:2706 A>G	22	7
ATPase6	chrM:8860 A>G	33	10
ATTCR:CR	chrM:16519 T>C	21	6
COI	chrM:7028 C>T	20	8
ND2	chrM:4769 A>G	23	7
ND4	chrM:11719 G>A	20	6

## Data Availability

The original contributions presented in this study are included in the article/Supplementary Materials. Further inquiries can be directed at the corresponding authors. Raw sequencing data is available upon request.

## Supplementary Materials

The following supporting information can be downloaded at: https://www.mdpi.com/article/10.3390/cells15060527/s1, Supplementary File S1; Supplementary File S2; Supplementary File S3; Supplementary File S4 [17,27,28,29,30,59,61,68,75,76,77,78,79]; Supplementary File S5.
