# Supplementary material for: Distinct Mutation Signatures in Peripheral Blood Mitochondrial DNA from Liquid Biopsy Reveal Insights into Pancreatic Cancer
Source: Cells. 2026 Mar 16;15(6):527. doi: 10.3390/cells15060527 (PMC13025575; doi:10.3390/cells15060527)
Supplement: Supplementary file 1 [file cells-15-00527-s001.zip › Supplementary File S4.pdf]

Supplementary File 4

Correlations between mutational signatures and clinical parameters

MtDNA copy number

Comparison of PC patients and healthy controls revealed no significant difference in mean plasma mtDNA copy number (Fig. 1A), indicating similar circulating mtDNA levels at the cohort level. Because plasma mtDNA originates from multiple tissues, including circulating blood cells and potentially tumor-derived material, these measurements reflect a composite signal rather than mitochondrial content within tumor tissue. Despite similar group means, plasma mtDNA copy number showed markedly greater variability among PC patients than controls (Fig. 1A), indicating substantial inter-individual heterogeneity within the cancer cohort. Increased dispersion of circulating mtDNA has been reported in cancer and other conditions associated with systemic cellular stress or tissue turnover [1–4]. To examine the relationship between mtDNA abundance and mutational patterns, plasma mtDNA copy number was analyzed relative to the AF of mtDNA SNVs. No linear correlation between mtDNA copy number and SNV AF was observed. However, stratification by plasma mtDNA copy number showed that patients in the higher-copy-number group (mean 87,085; SD 28,840; n = 15) had a significantly higher incidence of high-AF SNVs than those in the lower-copy-number group (mean 42,531; SD 18,863; n = 18) (Fig. 1C, p = 0.0043). Clinical outcome analysis further indicated shorter OS in patients with higher plasma mtDNA copy numbers (p = 0.0251, Fig. 1D). No significant differences between groups were observed for age (p = 0.54), total SNVs (p = 0.52), unique SNVs (p = 0.70), or cancer-specific SNVs (p = 0.50).

These findings should be interpreted cautiously given the limited sample size and cohort stratification and require validation in larger studies incorporating tumor-specific mtDNA profiling. The results must also be considered in their methodological context. In a previous study [2], we systematically evaluated preanalytical variables affecting mitochondrial DNA (mtDNA) and nuclear DNA (nDNA) quantification in whole blood and plasma and demonstrated that mtDNA yield and mtDNA-to-nDNA ratios are highly sensitive to factors such as extraction method, storage temperature, tube type, and storage duration. These observations indicate that mtDNA copy number estimates derived from sequencing coverage can be influenced by technical variables and therefore require strict procedural standardization to ensure interpretability. In the present cohort, genomic nDNA was not independently isolated or quantified, which precluded normalization to the biologically preferred mtDNA-to-nDNA ratio. Alternative normalization strategies, including normalization to autosomal sequencing depth, spike-in controls, or orthogonal quantification using qPCR or digital PCR, were considered but could not be implemented because of the retrospective study design and prior sample processing. As

described in the Methods, mtDNA copy number was estimated from sequencing coverage using a fixed scaling constant (100) introduced solely for computational normalization. This constant does not assume 100 mtDNA copies per cell but converts sequencing depth into a relative measure of mtDNA abundance under standardized analytical conditions. Consequently, reported values reflect relative differences between samples rather than absolute mtDNA copies per cell or total cellular mtDNA content. While this approach enables robust intra-cohort comparisons, it limits cross-study comparability and precludes absolute biological interpretation. Future studies incorporating matched nuclear normalization and independent quantitative validation will be required to refine the biological and clinical interpretation of mtDNA copy number estimates.

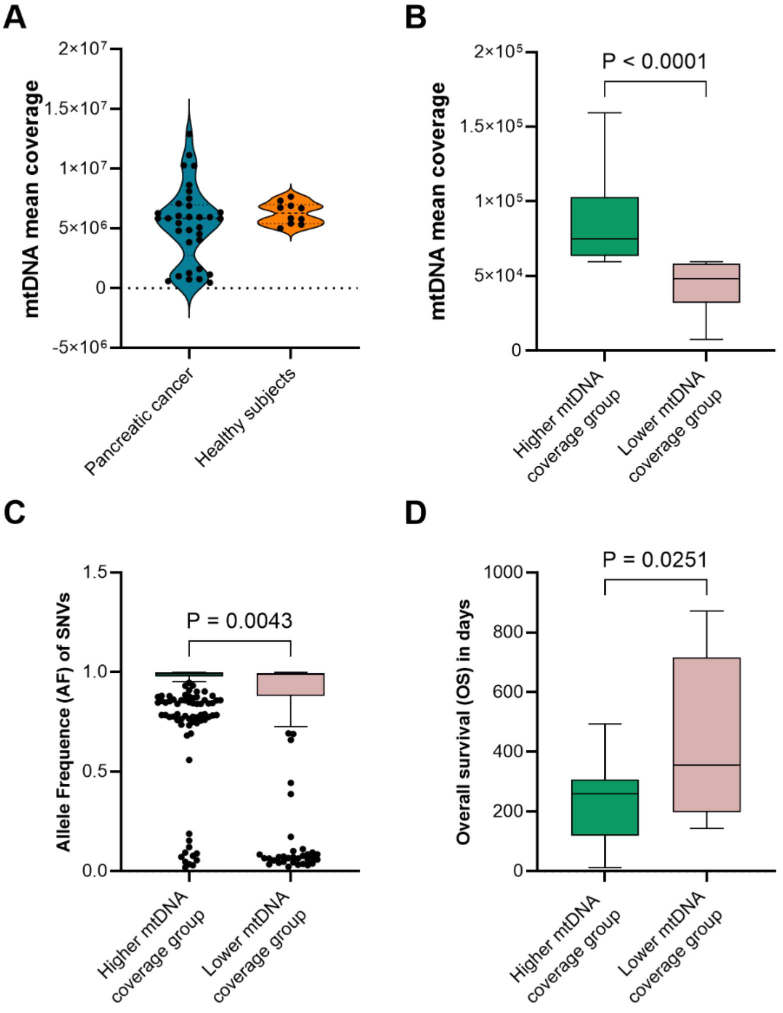

Figure S1. Assessment of mtDNA copy number and clinical implications in pancreatic cancer. (A) Violin plot comparing relative mtDNA copy number in whole blood between PC patients (n = 33) and healthy controls (n = 10). While the average mtDNA copy number does not significantly differ between groups, PC patients exhibit greater variability. (B) Box plot showing mtDNA copy number stratified by coverage group within PC patients, with a significant difference ($P < 0.0001$) between higher (n = 15) and lower (n = 18)-mtDNA-coverage groups. (C)

Box plot of SNV AFs across higher- and lower-mtDNA-coverage groups in PC patients, showing significantly elevated AF in the lower-mtDNA-coverage group ($P = 0.0043$). (D) OS in days for PC patients based on mtDNA coverage groups, with lower mtDNA coverage associated with improved survival ($P = 0.0251$), suggesting a potential link between mtDNA abundance and prognosis in pancreatic cancer. The latter survival analysis is exploratory in nature, as it is based on cohort stratification and limited subgroup sizes. Non-significant differences in mean mtDNA copy number (A) should be interpreted cautiously due to limited power; variability patterns and subgroup associations are exploratory and require validation. All group comparisons were performed using two-tailed unpaired t-tests.

3.4.2. SNV abundance and distribution

The total number of mtDNA SNVs showed no significant linear correlation with clinical parameters including age, sex, EGFR therapy, OS, mtDNA copy number, mean AF, or the number of low-AF SNVs. However, total SNV count per patient showed a weak positive correlation with the number of unique SNVs (Pearson $r = 0.4898$, $p = 0.0038$) (Suppl. Fig. 1), a moderate correlation with cancer-specific SNVs detected only in the cancer cohort ($r = 0.59$, $p = 0.0003$) (Suppl. Fig. 2), and a strong correlation with SNVs also present in the healthy population ($r = 0.87$, $p < 0.0001$) (Suppl. Fig. 3). These findings indicate that variation in total mtDNA SNV counts across patients was primarily driven by variants shared with the healthy population, whereas associations with unique or cancer-specific variants were weaker.

When patients were stratified according to the abundance of unique mtDNA SNVs ($p < 0.0001$, Fig. 2A), several differences between groups were observed. Among patients with available OS data, those with a higher number of unique SNVs ($n = 12$) showed longer OS than patients with fewer unique SNVs ($n = 11$), although this difference did not reach statistical significance in the initial analysis ($p = 0.11$). After exclusion of one outlier, the difference reached statistical significance ($p = 0.03$, Fig. 2B). Because the result was sensitive to the presence of a single observation, this association should be interpreted cautiously. Patients with a higher number of unique SNVs ($n = 15$) also showed a greater number of SNVs with an $AF > 0.7$ compared with patients with fewer unique SNVs ($n = 18$), approaching statistical significance ($p = 0.055$, Fig. 2C).

No linear correlations were detected between SNV AF and other clinical or genetic parameters. To further evaluate variant frequency patterns, patients were stratified into two AF groups (Group 1: AF 0.48–0.916, mean = 0.85, $n = 17$; Group 2: AF 0.918–0.97, mean = 0.95, $n = 16$). The higher-AF group showed a higher mtDNA copy number than the lower-AF group, although this difference was not statistically significant ($p = 0.288$, Fig. 2D). In contrast, the proportion of cancer-specific SNVs was significantly higher in the higher-AF group ($p = 0.016$, Fig. 2E). No significant differences between

groups with higher versus lower unique SNV abundance were observed for age (p = 0.2), mean SNV AF (p = 0.45), or mtDNA sequencing coverage (p = 0.69). Previous studies have reported associations between higher nuclear and mitochondrial mutation loads and improved responses to immunotherapy in certain cancer types [5,6].

When patients were stratified by the abundance of cancer-specific SNVs (p < 0.0001) (Suppl. Fig. 4), no statistically significant differences in clinical variables were observed between high and low groups. Patients with higher numbers of cancer-specific SNVs showed a non-significant trend toward longer OS (p = 0.38) (Suppl. Fig. 5) and higher mtDNA copy number (p = 0.24) (Suppl. Fig. 1). Individuals with more unique SNVs also tended to have a higher proportion of cancer-specific SNVs (p = 0.08, Fig. 2H), and total unique SNVs were moderately correlated with cancer-specific SNVs (r = 0.65, p = 0.0001) (Suppl. Fig. 2). These findings indicate partial overlap between unique and cancer-specific mtDNA variant patterns within the cohort.

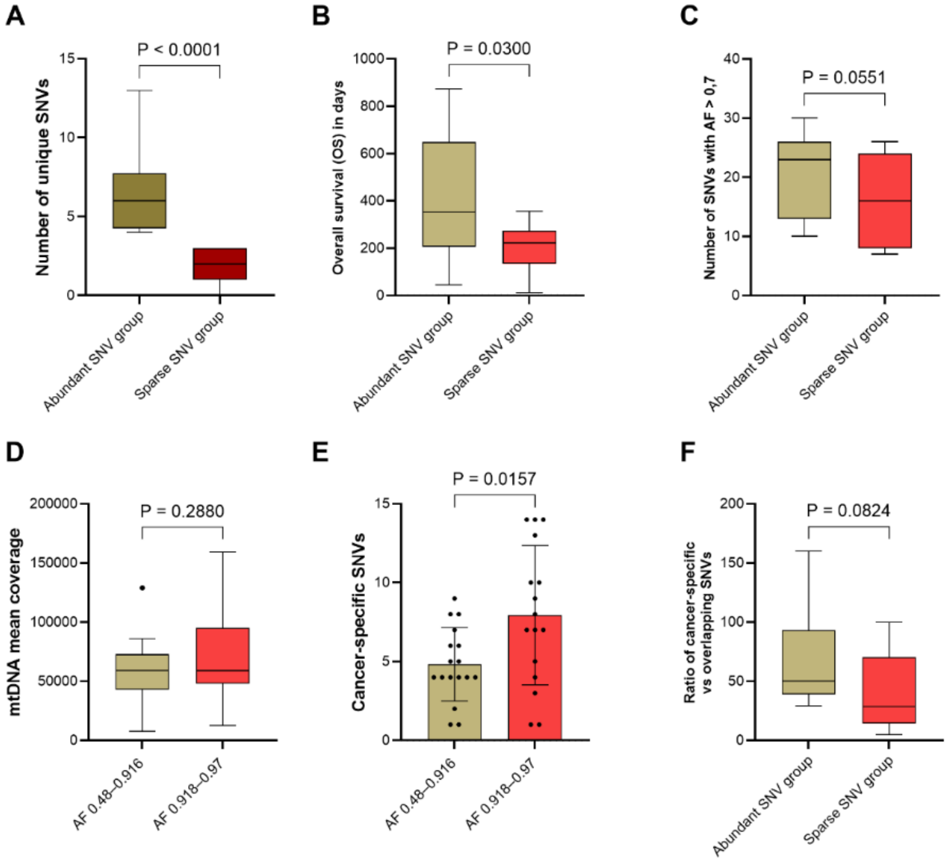

Figure S2. Associations between SNV characteristics and clinical parameters in PC patients. Patients were stratified by unique SNV abundance into high ("abundant," n = 12) and low ("sparse," n = 11) groups. (A) The abundant group had significantly more unique SNVs (p < 0.0001). (B) OS was longer in the abundant group (p = 0.03;

exploratory analysis, small subgroups). (C) SNVs with AF > 0.7 showed a near-significant difference between groups (p = 0.055). (D) mtDNA mean coverage did not differ between AF-defined groups (0.48–0.916 vs. 0.918–0.97; p = 0.288). (E) Cancer-specific SNVs (only found in cancer cohort) were more frequent in the higher-AF group (p = 0.016). (F) The ratio of cancer-specific to overlapping SNVs was higher in the abundant group, without reaching significance (p = 0.082). All comparisons were performed using two-tailed unpaired t-tests; subgroup analyses are exploratory and require validation in larger cohorts.

3.4.3. Patient characteristics

3.4.3.1. Age and sex

The analysis did not reveal a statistically significant linear correlation between age and any clinical parameters. Except for AF distribution, there were no statistically significant differences in clinical parameters between male and female patients. Specifically, male patients exhibited significantly higher levels of heteroplasmy than females (P = 0.0136; Suppl. Fig. 3), which may underlie certain sex-specific effects observed in mitochondrial diseases [7]. This difference could be attributed to factors such as increased mitochondrial ROS production in male skeletal muscle [8] or hormonal influences. These findings show the importance of accounting for sex differences in mitochondrial biology to better understand disease mechanisms and potential therapeutic approaches [8,9].

3.4.3.2. Overall survival (OS)

No consistent linear relationship was observed between OS and the clinical variables assessed. However, when considered alongside the mtDNA and SNV analyses presented earlier, several variables differed when patients were stratified by survival. As shown in the mtDNA copy number analysis (Fig. 1D), patients with higher plasma mtDNA copy numbers had shorter OS within this cohort. Previous studies have reported associations between elevated mtDNA levels and metabolic adaptations in tumors, including increased reliance on oxidative phosphorylation under hypoxic conditions [6,10,11]. However, tumor metabolism was not assessed in the present study, and the observed association between mtDNA copy number and OS should therefore be interpreted as descriptive rather than causal. The SNV diversity analysis (Fig. 2) further showed that patients with a higher number of unique mtDNA SNVs tended to have longer OS, whereas patients with fewer unique SNVs showed shorter OS (Fig. 2B). As noted previously, the statistical significance of this observation was sensitive to the treatment of an individual outlier and should therefore be interpreted cautiously. Studies in other cancer settings have reported associations between higher mutation burdens and treatment response or clinical outcome [12,13]. However, the functional impact of the mtDNA SNVs identified in this cohort was not assessed, and their potential clinical relevance remains uncertain.

Additional patterns emerged in relation to variant frequency. Patients with higher mtDNA coverage tended to show a greater number of high-AF SNVs (Fig. 1C), whereas patients with fewer unique SNVs and shorter OS displayed a higher frequency of low-AF variants. Overall, these observations describe differences in mtDNA variant distribution across survival groups within the cohort.

No statistically significant association between age and OS was detected when age was analyzed in two groups. However, when OS was stratified into two survival categories (Fig. 3A), Group A (11–276 days; mean = 174; SD = 86; n = 12) and Group B (283–872 days; mean = 494; SD = 205; n = 12), age was marginally higher in Group A, corresponding to shorter OS, although this difference did not reach statistical significance (p = 0.086) (Fig. 3B). These analyses describe associations between mtDNA-related variables, SNV characteristics, and OS within this cohort. Because of the exploratory design, limited sample size, and absence of multivariable modelling, the observed patterns should be interpreted cautiously and require validation in larger studies.

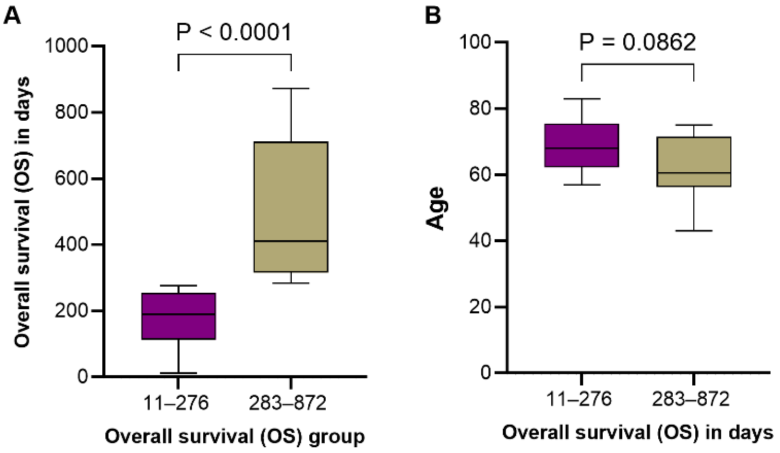

Figure S3. Comparison of OS and age between two patient groups with different OS durations. (A) Overall survival in days for PC patients stratified into a short OS group (11–276 days, n = 12) and a long OS group (283–872 days, n = 12). Group means were compared using a two-tailed unpaired t-test, revealing a significant difference (P < 0.0001). (B) Age distribution in the same short and long OS groups (n = 12 per group), compared using a two-tailed unpaired t-test, showing a higher mean age in the short OS group that did not reach statistical significance (P = 0.0862).

Methodological considerations not discussed in main text:

Data analysis: In the absence of matched tumor or longitudinal samples, mtDNA variants were operationally classified based on their distribution across individuals and allele frequency (AF), with shared or high-frequency variants interpreted as germline or polymorphic and low-frequency, individual-specific variants as putative

somatic events. AF groupings were guided by the observed bimodal distribution of mtDNA variants (Main Fig. 2 and Suppl. Fig. 1B). Accordingly, variants were categorized using predefined thresholds, with low-frequency heteroplasmic variants defined as AF < 0.7 and high-frequency, near-homoplasmic variants as AF ≥ 0.7. Patient stratification used empirically observed AF ranges (0.48–0.916 and 0.918–0.97; Suppl. Fig. 1B and Fig. 2D).

Statistical analysis and limitations: Comparisons between two independent groups were performed using two-tailed unpaired tests, with the choice of parametric or non-parametric testing guided by visual assessment of data distribution. Linear associations were evaluated using linear regression, and correlation strength was quantified using the Pearson correlation coefficient. OS was analyzed as a continuous variable and compared between groups using two-tailed unpaired tests. Grubbs' test was applied to identify potential outliers where indicated. All statistical tests were two-sided, and p values less than 0.05 were considered statistically significant. Exact sample sizes, grouping criteria, and statistical tests applied to individual comparisons are specified in the corresponding figure legends. Continuous variables were initially examined for linear associations with clinical outcomes using correlation and regression analyses. As no consistent linear relationships were observed, selected continuous variables were subsequently stratified into discrete groups using predefined or data-driven thresholds to enable comparative analyses. This strategy was applied to explore potential non-linear associations and subgroup effects that may not be captured by linear models, particularly in the context of biological heterogeneity. Accordingly, group-based comparisons were used as an exploratory approach to identify patterns potentially associated with clinical outcomes, and survival analyses shown in Figs. 1D and 2B should therefore be interpreted as exploratory, as they are based on cohort stratification into small subgroups and, in the case of Fig. 2B, are sensitive to the exclusion of a single outlier.

Power considerations: This study was designed as an exploratory, hypothesis-generating analysis of whole-blood mtDNA features in PC. Given the cohort size of 33 patients and 10 healthy controls, formal a priori power calculations were not feasible. Post hoc considerations indicate that, for two-group comparisons at a two-sided α = 0.05 with 80% power, the study is adequately powered primarily to detect large standardized effects, with a minimum detectable Cohen's d of approximately 1.0. Consequently, non-significant between-group results, such as those in main Fig. 1A and Suppl. Figure 1A, should not be interpreted as evidence of no difference, as moderate or small effects may not be reliably detectable. This applies both to case–control comparisons (Main Fig. 1 and Suppl. Fig. 1A; n = 33 vs n = 10) and to within-patient sub-group analyses (Fig. 1C; n = 15 vs n = 18). Survival analyses (Figs. 1D and 2B) are further constrained by small subgroup sizes and event counts; under near-complete follow-up, detectable hazard ratios are approximately 2.7 for Fig. 1D and 3.2 for Fig. 2B, with larger effects required in the presence of censoring. Accordingly, survival-related associations should be regarded as hypothesis-generating and require validation in larger cohorts.

References

1. Kumar, M.; Srivastava, S.; Singh, S.A.; Das, A.K.; Das, G.C.; Dhar, B.; Ghosh, S.K.; Mondal, R. Cell-Free Mitochondrial DNA Copy Number Variation in Head and Neck Squamous Cell Carcinoma: A Study of Non-Invasive Biomarker from Northeast India. *Tumour Biol. J. Int. Soc. Oncodevelopmental Biol. Med.* **2017**, *39*, 1010428317736643. https://doi.org/10.1177/1010428317736643.

2. Randeu, H.; Bronkhorst, A.J.; Mayer, Z.; Oberhofer, A.; Polatoglou, E.; Heinemann, V.; Haas, M.; Boeck, S.; Holdenrieder, S. Preanalytical Variables in the Analysis of Mitochondrial DNA in Whole Blood and Plasma from Pancreatic Cancer Patients. *Diagnostics* **2022**, *12*, 1905. https://doi.org/10.3390/diagnostics12081905.

3. Smith, A.L.M.; Whitehall, J.C.; Greaves, L.C. Mitochondrial DNA Mutations in Ageing and Cancer. *Mol. Oncol.* **2022**, *16*, 3276–3294. https://doi.org/10.1002/1878-0261.13291.

4. Yuan, Y.; Ju, Y.S.; Kim, Y.; Li, J.; Wang, Y.; Yoon, C.J.; Yang, Y.; Martincorena, I.; Creighton, C.J.; Weinstein, J.N.; et al. Comprehensive Molecular Characterization of Mitochondrial Genomes in Human Cancers. *Nat. Genet.* **2020**, *52*, 342–352. https://doi.org/10.1038/s41588-019-0557-x.

5. Samstein, R.M.; Lee, C.-H.; Shoushtari, A.N.; Hellmann, M.D.; Shen, R.; Janjigian, Y.Y.; Barron, D.A.; Zehir, A.; Jordan, E.J.; Omuro, A.; et al. Tumor Mutational Load Predicts Survival after Immunotherapy across Multiple Cancer Types. *Nat. Genet.* **2019**, *51*, 202–206. https://doi.org/10.1038/s41588-018-0312-8.

6. Mahmood, M.; Liu, E.M.; Shergold, A.L.; Tolla, E.; Tait-Mulder, J.; Huerta Uribe, A.; Shokry, E.; Young, A.L.; Lilla, S.; Kim, M.; et al. Mitochondrial DNA Mutations Drive Aerobic Glycolysis to Enhance Checkpoint Blockade Response in Melanoma. *Nat. Cancer* **2024**. https://doi.org/10.1038/s43018-023-00721-w.

7. Wang, L.; Cheng, H.-X.; Zhou, Y.-H.; Ma, M. Clinical Significance of the D-Loop Gene Mutation in Mitochondrial DNA in Laryngeal Cancer. *OncoTargets Ther.* **2021**, *14*, 3461–3466. https://doi.org/10.2147/OTT.S304836.

8. Junker, A.; Wang, J.; Gouspillou, G.; Ehinger, J.K.; Elmér, E.; Sjövall, F.; Fisher-Wellman, K.H.; Neufer, P.D.; Molina, A.J.A.; Ferrucci, L.; et al. Human Studies of Mitochondrial Biology Demonstrate an Overall Lack of Binary Sex Differences: A Multivariate Meta-Analysis. *FASEB J.* **2022**, *36*, e22146. https://doi.org/10.1096/fj.202101628R.

9. Ventura-Clapier, R.; Moulin, M.; Piquereau, J.; Lemaire, C.; Mericskay, M.; Veksler, V.; Garnier, A. Mitochondria: A Central Target for Sex Differences in Pathologies. *Clin. Sci.* **2017**, *131*, 803–822. https://doi.org/10.1042/CS20160485.

10. Mambo, E.; Chatterjee, A.; Xing, M.; Tallini, G.; Haugen, B.R.; Yeung, S.-C.J.; Sukumar, S.; Sidransky, D. Tumor-Specific Changes in mtDNA Content in Human Cancer. *Int. J. Cancer* **2005**, *116*, 920–924. https://doi.org/10.1002/ijc.21110.

11. van Kraaij, S.J.W.; Pereira, D.R.; Smal, B.; Summo, L.; Konkel, A.; Lossie, J.; Busjahn, A.; Grammatopoulos, T.N.; Klaassen, E.; Fischer, R.; et al. Identification of Peripheral Vascular Function Measures and Circulating Biomarkers of Mitochondrial Function in Patients with Mitochondrial Disease. *Clin. Transl. Sci.* **2023**, *16*, 1258–1271. https://doi.org/10.1111/cts.13530.

12. Rizvi, N.A.; Hellmann, M.D.; Snyder, A.; Kvistborg, P.; Makarov, V.; Havel, J.J.; Lee, W.; Yuan, J.; Wong, P.; Ho, T.S.; et al. Mutational Landscape Determines Sensitivity to PD-1 Blockade in Non–Small Cell Lung Cancer. *Science* **2015**, *348*, 124–128. https://doi.org/10.1126/science.aaa1348.

13. Hopkins, J.F.; Denroche, R.E.; Aguiar, J.A.; Notta, F.; Connor, A.A.; Wilson, J.M.; Stein, L.D.; Gallinger, S.; Boutros, P.C. Mutations in Mitochondrial DNA From Pancreatic Ductal Adenocarcinomas Associate With Survival Times of Patients and Accumulate as Tumors Progress. *Gastroenterology* **2018**, *154*, 1620–1624.e5. https://doi.org/10.1053/j.gastro.2018.01.029.
