# Supplementary material for: Distinct Mutation Signatures in Peripheral Blood Mitochondrial DNA from Liquid Biopsy Reveal Insights into Pancreatic Cancer
Source: Cells. 2026 Mar 16;15(6):527. doi: 10.3390/cells15060527 (PMC13025575; doi:10.3390/cells15060527)
Supplement: Supplementary file 1 [file cells-15-00527-s001.zip › Supplementary File S5.pdf]

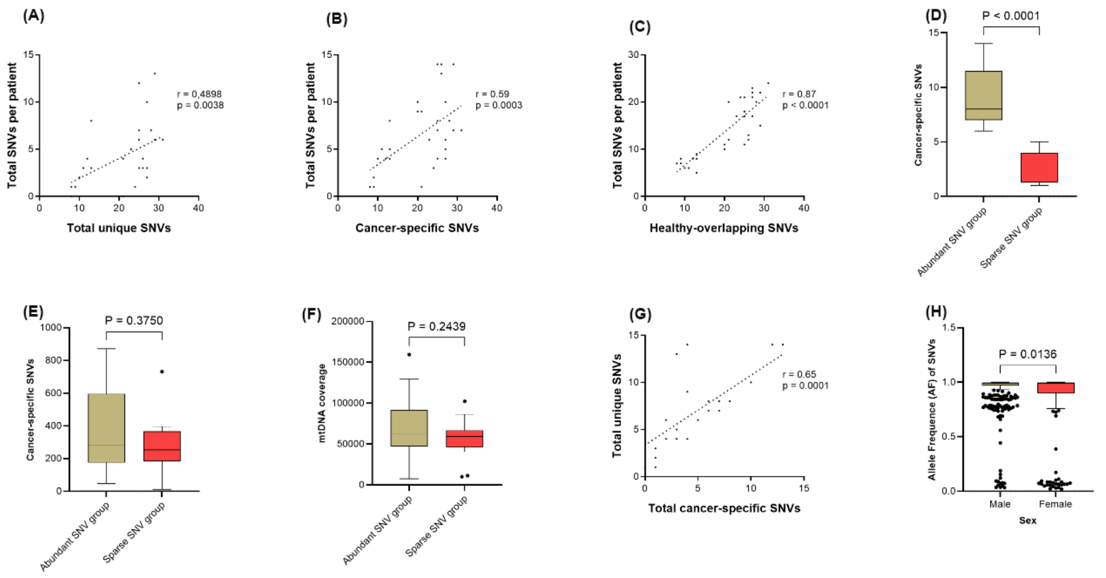

Supplementary Figure S4. (A–H) This figure summarizes the relationships between mtDNA SNV burden, SNV subcategories, and clinical or biological features in pancreatic cancer patients. The total number of SNVs per patient showed a weak positive correlation with the total number of unique SNVs (**A**; Pearson $r = 0.4898$, $p = 0.0038$), a moderate correlation with cancer-specific SNVs (**B**; $r = 0.59$, $p = 0.0003$), and a strong correlation with SNVs overlapping those found in the healthy population (**C**; $r = 0.87$, $p < 0.0001$), indicating that overall mutation burden is largely driven by common, non-cancer-associated variants. Patients stratified by the abundance of unique SNVs showed a significantly higher number of cancer-specific SNVs in the abundant SNV group compared with the sparse SNV group (**D**; $p < 0.0001$). However, no statistically significant differences were observed between abundant and sparse cancer-specific SNV groups with respect to overall survival (**E**; $p = 0.3750$) or mtDNA coverage (**F**; $p = 0.2439$). A moderate positive correlation was observed between total unique SNVs and total cancer-specific SNVs (**G**; $r = 0.65$, $p = 0.0001$), supporting an association between unique mtDNA variation and cancer-related mutations. Finally, allele frequency analysis revealed significantly higher heteroplasmy levels in male patients compared with female patients (**H**; $p = 0.0136$), highlighting sex-specific differences in mitochondrial genetic variation.
